# Association between weight-adjusted waist index and 20-meter shuttle run test in Chinese children and adolescents: a multicenter cross-sectional survey

**DOI:** 10.3389/fpubh.2025.1550741

**Published:** 2025-04-22

**Authors:** Zhen Zhang, Nana Tang, Mengjin Yao, Zhimin Zhao

**Affiliations:** ^1^School of Physical Education, Huanghuai University, Zhumadian, China; ^2^School of Physical Education and Sports, Shihezi University, Shihezi, China

**Keywords:** children and adolescents, weight-adjusted waist index, cardiopulmonary fitness, associations, cross-sectional survey 1 introduction

## Abstract

**Background:**

Cardiopulmonary fitness is associated with several physical health indicators in children and adolescents and has shown a downward trend in recent years. The 20-m SRT has received widespread attention from scholars as a recognized indirect measure for evaluating cardiopulmonary fitness. However, few studies have analyzed the association between the weight-adjusted waist index (WWI), a novel indicator, and cardiopulmonary fitness in Chinese children and adolescents.

**Methods:**

In this study, 41,523 children and adolescents aged 6–17 years in China were assessed for weight, waist circumference, 20-m SRT, and related covariates. One-way ANOVA, LSD, and curvilinear regression analyses were used to analyze the associations that existed between WWI and 20-m SRT.

**Results:**

Comparison of WWI and 20-m SRT scores among Chinese children and adolescents of different ages showed statistically significant differences (*F*-values of 2179.297 and 4956.795, respectively, *p* < 0.001). Overall, the 20-m SRT scores of both boys and girls showed a general trend of increasing with age, with a maximum of 48.78 laps and 31.17 laps in the age group of 15–17 years, respectively. The differences in 20-m SRT scores were statistically significant (*p* < 0.001) when compared between different WWI groups. Both lower and higher WWI resulted in lower 20-m SRT scores, more significantly in boys compared to girls.

**Conclusion:**

There is an inverted “U” curve relationship between WWI and 20-m SRT in Chinese children and adolescents aged 6–17 years, and this relationship is reflected in different genders and age groups. In the future, we should ensure that the WWI of children and adolescents is within a reasonable range to better promote cardiopulmonary fitness.

## 1 Introduction

Cardiopulmonary fitness is an important factor in the physical health of children and adolescents, with a significance and impact on their physical and mental health, academic performance, executive functioning, and future achievement (1–3). The 20-m SRT is currently the most commonly used assessment of cardiopulmonary fitness in children and adolescents worldwide (4). A study of 20-m SRT in children and adolescents from more than 50 countries around the world showed a strong association between cardiopulmonary fitness, as assessed by 20-m SRT, and several health outcomes (5). Multiple studies have confirmed that reduced cardiopulmonary fitness is associated with increased mental health risk, decreased physical fitness, and decreased academic performance (6–8). It has been found that increased cardiopulmonary fitness helps to increase the elasticity and function of the body’s blood vessels, lowering blood lipid concentrations and increasing capillary density, thereby improving vascular health (9). It has also been shown that cardiopulmonary fitness is negatively associated with the risk of developing metabolic syndrome, including hypertension, hyperglycemia, dyslipidemia, and obesity (10). An analysis of a cohort of 750,302 people found that participants with the worst cardiopulmonary fitness were significantly associated with a 309% increased risk of death (11, 12). However, with the constant changes in modern lifestyles, such as the decline in physical activity levels, the prolongation of static behaviors, the increase in the time spent on video screen behaviors, and the occurrence of obesity problems, all of which are important reasons for the decline in cardiopulmonary fitness among children and adolescents from year to year (13). Studies have shown a positive correlation between decreased levels of physical activity and decreased cardiopulmonary fitness (14). It has also been shown that obese children and adolescents have lower levels of cardiopulmonary fitness compared to normal-weight individuals (15). This shows that there is a strong association between fat content and cardiopulmonary fitness. In addition, it has also been found that cardiopulmonary fitness is significantly associated with physical activity and that fat mass is negatively associated with cardiopulmonary fitness (16). It can be seen that the causes of the decline in cardiopulmonary fitness among children and adolescents are manifold, and the main reasons affecting the decline in cardiopulmonary fitness among adolescents should be discovered through investigation and research, and appropriate interventions should be carried out according to the reasons, to better improve the level of cardiopulmonary fitness among children and adolescents. The fitness level of children and adolescents.

In recent years, WWI as a new type of index for assessing body obesity in children and adolescents has received extensive attention from scholars.WWI is an important index for assessing body composition, which eliminates the influence of body weight on body composition, and its assessment results are more objective and real. A study comparing the relationship between eight body fat metrics, including BMI, waist circumference, waist-to-hip ratio, waist-to-height ratio, and WWI, and the risk of diabetes, cardiovascular disease, and nonaccidental death found that WWI had the most robust and consistent association with cardiovascular disease and nonaccidental death risk (17). It has also been shown that when the WWI is higher than 11.2, there is a significant association between cardiovascular disease and all-cause mortality and that it is an effective predictor of cardiovascular disease (18). It can be seen that WWI has significant effects and advantages in assessing the level of human health. However, with the change of lifestyle, the intake of high-energy food has led to the seriousness of the problem of obesity and the trend of increase, which will inevitably lead to the increasing value of WWI, and thus negatively affect the development of physical and mental health (19). However, several past studies have focused on the relationship between conventional BMI, waist circumference, and other indicators and health outcomes in children and adolescents, while fewer studies have addressed the relationship between WWI and related health indicators (20). For this reason, future studies should be conducted on WWI to analyze the associations that exist with health outcomes.

WWI is a novel indicator for evaluating body composition in recent years, and its related studies are relatively few. What kind of relationship exists between 20-m SRT, an important indicator for evaluating cardiopulmonary fitness in children and adolescents, and WWI needs to be further investigated and studied. In addition, past studies have found even fewer studies on the relationship between WWI and related physical fitness indicators in children and adolescents (21). Moreover, past research on WWI and physical fitness indicators has mainly focused on children and adolescents in Western developed countries, and there are few studies on Chinese children and adolescents at the national level (22). In this study, 41,523 children and adolescents aged 6–17 years from five regions of China were assessed for WWI and 20-m SRT, to analyze the association between WWI and cardiopulmonary fitness in Chinese children and adolescents, and to provide necessary support and assistance for the development of physical fitness and interventions for Chinese children and adolescents.

## 2 Methods

### 2.1 Participants

**Figure 1:**
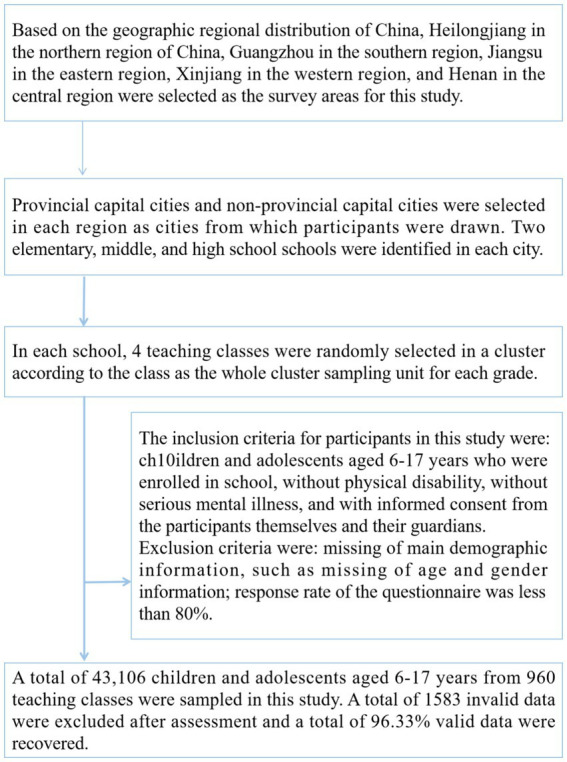
Participant sampling process.

In this study, 41,523 children and adolescents aged 6–17 years from different regions of China were assessed for the WWI and 20-m SRT indicators using a three-stage stratified whole-cluster random sampling method. The specific participant sampling process was as follows. First, based on the geographic regional distribution of China, Heilongjiang in the northern region of China, Guangzhou in the southern region, Jiangsu in the eastern region, Xinjiang in the western region, and Henan in the central region were selected as the survey areas for this study. Second, provincial capital cities and non-provincial capital cities were selected in each region as cities from which participants were drawn. Two elementary, middle, and high school schools were identified in each city. Third, in each school, 4 teaching classes were randomly selected in a cluster according to the class as the whole cluster sampling unit for each grade. A total of 43,106 children and adolescents aged 6–17 years from 960 teaching classes were sampled in this study. A total of 1,583 invalid data were excluded after assessment and a total of 96.33% valid data were recovered. The inclusion criteria for participants in this study were: children and adolescents aged 6–17 years who were enrolled in school, without physical disability, without serious mental illness, and with informed consent from the participants themselves and their guardians. Exclusion criteria were: missing of main demographic information, such as missing of age and gender information; response rate of the questionnaire was less than 80%. The specific process of sampling participants for this study is shown in Figure 1.

This study was conducted by the Declaration of Helsinki. Informed consent was obtained from parents or guardians before the assessment of participants in this study, and participants volunteered to be assessed for this study. This study was approved by the Ethics Committee of Huanghuai University (415789542) in May 2023.

### 2.2 Weight-adjusted waist index (WWI)

The WWI was calculated based on participants’ weight and waist circumference assessments. The formula is waist circumference (cm) divided by the square root of weight (kg) (23). In this study, weight and waist circumference were assessed according to the assessment methods and instruments required by the China National Survey on Students’ Constitution and Health (CNSSCH) (24). Weight was assessed to the nearest 0.1 kg and waist circumference to the nearest 0.1 cm. Participants were asked to empty their bowels before weight and waist circumference assessments and to wear light clothing for the assessments. Waist circumference was assessed by staff of the same sex. In this study, after stratifying the participants’ WWI based on age and gender, they were categorized into 5 equal scores according to percentile to compare the relationship between different WWI levels and 20-m SRT scores.

### 2.3 20-meter shuttle run test (20-m SRT)

The 20-m SRT is widely used as an indirect assessment of cardiopulmonary fitness in children and adolescents in most countries worldwide (25). The 20-m SRT test developed by Cooper Laboratories in the United States was used in this study (26). The 20-m SRT was assessed by drawing a starting line at each end of a distance of 20 m. When the participant heard a beep from the CD player, he or she ran at the appropriate speed to the opposite end of the line, and when he or she heard the beep again from the SD player, he or she ran to the opposite end of the line, and so on back and forth, with each 20-m distance run being recorded as 1 lap (times). The speed at which the participant ran was controlled by the CD music, starting at an initial speed of 8.0 km/h, 9.0 km/h in the 2nd minute, and then accelerating by one-speed level per minute, i.e., increasing by 0.5 km/h each time. The tester did his/her best to complete the speed levels, and when he/she could not follow the tempo to reach the 20 m ending on 2 consecutive occasions, then the test was over, and the last laps were recorded. The complete 20-mSRT consists of 247 laps in 21 levels.

### 2.4 Quality control

All staff members of the Institute who participate in the assessment undergo rigorous assessment training and assessment before the assessment, and those who are qualified after the assessment participate in the assessment work. After the assessment of the participants, the staff will fill in the assessment results on the assessment cards of the participants, and the participants are required to strictly prohibit alteration. Staff members are required to calibrate the instruments and equipment used in the assessment before each day’s assessment, and the assessment can only be carried out after they have passed the calibration.

### 2.5 Statistical analysis

Height, weight, waist circumference, WWI, and 20-m SRT scores in this study were expressed using mean and standard deviation. The proportional distribution of the number of boys and girls in different age groups was expressed using percentages. Comparison of continuous variables between different age groups was done by using one-way method analysis. After WWI was stratified according to different ages and genders in this study, it was divided into five groups according to percentile: WWI <20th (A), 20th ≤ WWWI <40th (B), 40th ≤ WWWI <60th (C), 60th ≤ WWWI <80th (D), and WWI ≥80th (E). Comparisons between different WWI groups and 20-m SRT scores were compared using one-way ANOVA. Comparisons between different WWI groups were performed using LSD for between-group comparisons.

Based on past research experience, the association between WWI and 20-m SRT in children and adolescents in this study was analyzed using curvilinear regression. Curvilinear regression analysis was performed with laps of 20-m SRT as the dependent variable and WWI as the independent variable. To understand the association between WWI and 20-m SRT in different age groups, children and adolescents aged 6–17 years were divided into four groups in this study, which were 6–8 years, 9–11 years, 12–14 years, and 15–17 years, respectively. The regression equation was established for each age group based on the results of the curvilinear regression analysis, Y = aX^2^ + bX + c, where a, b, and c are the constant terms, Y denotes 20-m SRT, and X denotes WWI. Adjusted R^2^ values are reported for each equation.

The data in this study were analyzed using SPSS 25.0 software, and the pictures were created using Graph Pad Prism 8 software. *p* < 0.05 was used as a two-sided test level.

## 3 Results

In this study, 41,523 children and adolescents aged 6–17 years were assessed in five geographic regions of China, including 21,424 boys (51.6%) and 20,099 girls (48.4%). The mean age of the participants was (11.38 ± 3.46) years. The number of boys and girls in each age group is shown in Table 1.

**Table 1:** Distribution of the number of children and adolescents aged 6–17 by age group in China [*n* (%)].

Age (years)	Boys	Girls	Total
6 years	2,310 (53.7)	1988 (46.3)	4,298
7 years	1,537 (52.2)	1,408 (47.8)	2,945
8 years	1779 (49.7)	1797 (50.3)	3,576
9 years	1885 (52.8)	1,688 (47.2)	3,573
10 years	1874 (53.3)	1,645 (46.7)	3,519
11 years	1751 (53.5)	1,522 (46.5)	3,273
12 years	1,684 (52.4)	1,528 (47.6)	3,212
13 years	1722 (52.3)	1,572 (47.7)	3,294
14 years	1911 (51.6)	1795 (48.4)	3,706
15 years	1972 (50.5)	1936 (49.5)	3,908
16 years	1,639 (48.5)	1738 (51.5)	3,377
17 years	1,360 (47.9)	1,482 (52.1)	2,842
6–17 years	21,424 (51.6)	20,099 (48.4)	41,523

Table 2 shows the comparison of WWI and 20-m SRT scores of Chinese adolescents in different age groups. Overall, the differences in Height, Weight, Waist circumference, WWI, and 20-m SRT scores of Chinese adolescents in different age groups were statistically significant when compared to each other (*F*-values of 37,356.238, 18,420.620, 43,646.565, 2,179.297, and 4,956.795, respectively, *p* < 0.001). Overall, the 20-m SRT scores of boys and girls showed an increasing trend with increasing age and were highest at 48.78laps and 31.17laps in the age group of 15–17 years, respectively. The results of the analysis in terms of boys and girls are presented in Table 2.

**Table 2:** Comparison of WWI and 20-m SRT scores among Chinese adolescents of different ages.

	N	Height [M (SD)]	Weight [M (SD)]	Waist circumference [M (SD)]	WWI [M (SD)]	20-m SRT(laps) [M (SD)]
Boys						
6–8 years	5,626	132.06 ± 9.11	30.11 ± 7.64	57.30 ± 11.10	10.56 ± 1.82	17.96 ± 8.72
9–11 years	5,510	149.56 ± 10.02	42.39 ± 10.87	65.78 ± 10.90	10.22 ± 1.35	27.64 ± 12.61
12–14 years	5,317	168.69 ± 8.47	57.30 ± 12.29	70.88 ± 10.43	9.43 ± 1.11	43.63 ± 18.22
15–17 years	4,971	173.56 ± 6.27	63.37 ± 11.09	74.04 ± 9.74	9.34 ± 0.94	48.78 ± 19.82
*F*-value		26074.496	10754.901	2569.333	1036.521	4622.11
*p*-value		<0.001	<0.001	<0.001	<0.001	<0.001
Girls						
6–8 years	5,193	130.93 ± 8.88	28.01 ± 6.44	55.02 ± 9.21	10.51 ± 1.64	17.43 ± 7.93
9–11 years	4,855	149.72 ± 9.12	40.24 ± 9.42	62.81 ± 9.05	10.01 ± 1.25	23.85 ± 10.63
12–14 years	4,895	160.92 ± 5.94	50.89 ± 8.18	66.23 ± 8.17	9.33 ± 1.01	28.32 ± 13.84
15–17 years	5,156	161.95 ± 5.55	52.51 ± 7.16	67.06 ± 7.38	9.28 ± 0.88	31.17 ± 11.24
*F*-value		18723.553	10680.885	2155.914	1166.324	1505.559
*P*-value		<0.001	<0.001	<0.001	<0.001	<0.001
Total						
6–8 years	10,819	131.52 ± 9.02	29.10 ± 7.17	56.20 ± 10.30	10.54 ± 1.74	17.71 ± 8.35
9–11 years	10,365	149.63 ± 9.61	41.38 ± 10.28	64.39 ± 10.18	10.12 ± 1.31	25.86 ± 11.88
12–14 years	10,212	164.97 ± 8.33	54.23 ± 11.00	68.65 ± 9.70	9.38 ± 1.06	36.29 ± 17.98
15–17 years	10,127	167.65 ± 8.29	57.84 ± 10.77	70.49 ± 9.30	9.31 ± 0.91	39.81 ± 18.29
*F*-value		37356.238	18420.620	4364.565	2179.297	4956.795
*P*-value		<0.001	<0.001	<0.001	<0.001	<0.001

M ± SD, mean ± standard deviation.

Table 3 shows the comparison of 20-m SRT scores of Chinese children and adolescents aged 6–17 years with different WWIs. Overall, the differences in 20-m SRT scores were statistically significant when compared between different WWI groups (*p* < 0.001). Comparisons between the age groups were made using the LSD method, and the results showed that, except for the B/C group of 6–8 years, the A/E group of 9–11 years, the A/E group of 12–14 years, and the B/C and C/D groups, and the A/C group of 15–17 years, which had no significant differences, the differences were statistically significant when compared to each other (*p* < 0.05). For boys and girls, the comparison of results between groups of WWI at different ages is shown in Table 3.

**Table 3:** Comparison of 20-m SRT scores across WWI in Chinese children and adolescents aged 6–17 years old.

Age (yr)	WWI < 20th (A)		20th ≤ WWI < 40th (B)		40th ≤ WWI < 60th (C)		60th ≤ WWI < 80th (D)		WWI ≥ 80th (E)		*F*-value	*P*-value	LSD *^#^*									
	N	M (SD)	N	M (SD)	N	M (SD)	N	M (SD)	N	M (SD)			A/B	A/C	A/D	A/E	B/C	B/D	B/E	C/D	C/E	D/E
Boys																						
6–8 years	682	17.58 ± 8.39	277	20.69 ± 9.51	584	21.18 ± 9.18	1,463	19.69 ± 9.24	2,620	16.09 ± 7.78	75.673	<0.001	#	#	#	#	—	—	#	#	#	#
9–11 years	657	27.84 ± 13.88	719	31.23 ± 13.26	1,054	30.35 ± 13.27	1,558	27.74 ± 12.21	1,522	23.87 ± 10.50	63.427	<0.001	#	#	—	#	—	#	#	#	#	#
12–14 years	1,410	44.50 ± 18.82	1,396	45.92 ± 18.10	1,295	44.16 ± 17.63	830	41.71 ± 17.36	386	34.52 ± 17.11	33.849	<0.001	#	—	#	#	#	#	#	#	#	#
15–17 years	1,401	49.13 ± 20.37	1,582	50.98 ± 20.20	1,137	48.92 ± 19.17	643	45.46 ± 18.24	208	39.18 ± 17.02	22.073	<0.001	#	—	#	#	#	#	#	#	#	#
Girls																						
6–8 years	592	17.28 ± 7.86	293	19.99 ± 9.15	696	19.63 ± 8.14	1,439	18.70 ± 8.19	2,173	15.58 ± 7.06	62.714	<0.001	#	#	#	#	—	#	#	#	#	#
9–11 years	683	18.55 ± 13.00	811	26.59 ± 9.89	1,159	25.70 ± 9.71	1,209	24.62 ± 10.01	993	22.18 ± 9.60	76.978	<0.001	#	#	#	#	—	#	#	#	#	#
12–14 years	1,368	20.04 ± 13.67	1,480	32.25 ± 11.45	1,225	31.47 ± 11.76	609	31.73 ± 15.04	213	26.43 ± 14.55	208.431	<0.001	#	#	#	#	—	—	#	—	#	#
15–17 years	1,516	31.25 ± 11.34	1791	32.36 ± 11.44	1,138	31.10 ± 10.66	535	28.68 ± 11.18	176	26.34 ± 9.62	20.068	<0.001	#	—	#	#	#	#	#	#	#	#
Total																						
6–8 years	1,274	17.44 ± 8.14	570	20.33 ± 9.33	1,280	20.34 ± 8.66	2,902	19.20 ± 8.75	4,793	15.86 ± 7.46	134.541	<0.001	#	#	#	#	—	#	#	#	#	#
9–11 years	1,340	23.10 ± 14.21	1,530	28.77 ± 11.82	2,213	27.91 ± 11.77	2,767	26.37 ± 11.41	2,515	23.20 ± 10.18	93.508	<0.001	#	#	#	—	#	#	#	#	#	#
12–14 years	2,778	32.46 ± 20.53	2,876	38.89 ± 16.53	2,520	37.99 ± 16.34	1,439	37.48 ± 17.13	599	31.65 ± 16.69	65.447	<0.001	#	#	#	—	—	#	#	—	#	#
15–17 years	2,917	39.84 ± 18.60	3,373	41.09 ± 18.63	2,275	40.01 ± 17.88	1,178	37.84 ± 17.55	384	33.29 ± 15.49	19.975	<0.001	#	—	#	#	#	#	#	#	#	#

M ± SD, mean ± standard deviation. # indicates a significant difference for LSD intergroup comparisons, *P* < 0.05.

**Figure 2:**
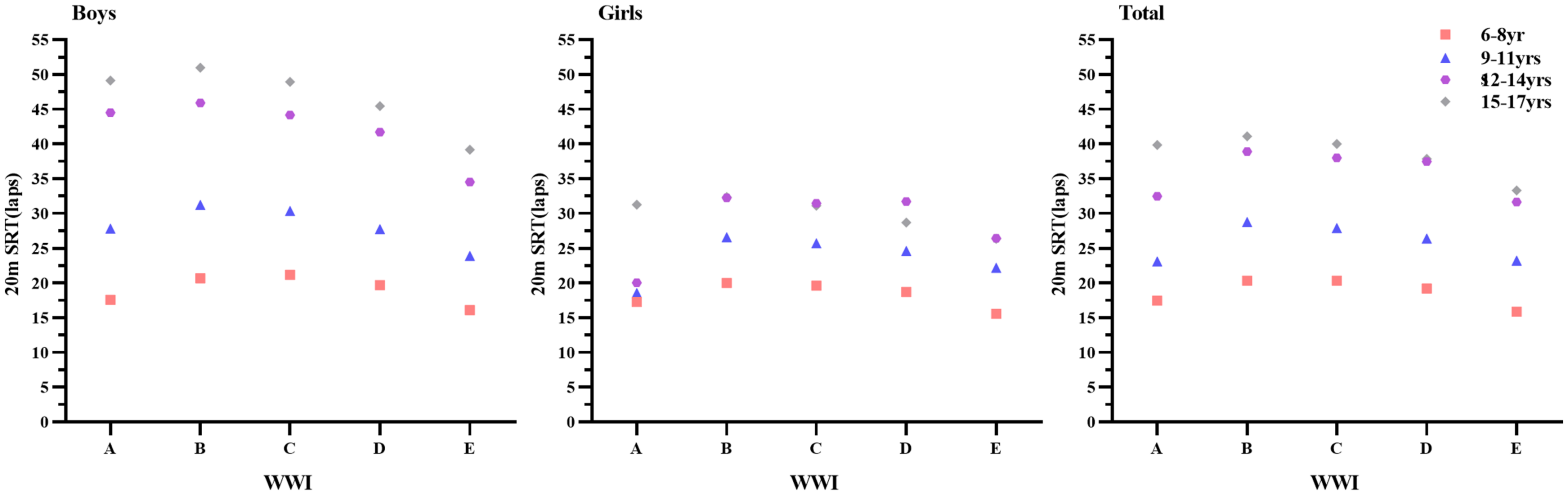
Trends in 20-m SRT scores of Chinese children and adolescents aged 6–17 years in different WWI subgroups. WWI < 20th **(A)**, 20th WWI < 40th **(B)**, 40th WWI < 60th **(C)**, 60th ≤ WWI < 80th **(D)**, WWI 80th **(E)**.

Figure 2 shows the trend of 20-m SRT scores of Chinese children and adolescents aged 6–17 years in different WWI groups. As can be seen from the figure, boys’ 20-m SRT scores in different WWI groups were generally higher than those of girls, and the trend of change was more pronounced compared with that of girls. Overall the 20-m SRT scores of boys and girls were highest when their WWI was in the range of group B. The 20-m SRT scores of boys and girls were higher than those of girls in different WWI groups.

In this study, the curvilinear regression analysis was performed by stratifying by gender, using 20-m SRT as the dependent variable (Y) and WWI as the independent variable (X), and the following curvilinear regression equation was derived:

Boys:


$$6–8\;\mathrm{years},Y=-0.210\times^{2}+3.571X+4.315\;R^{2}=0.031\text{.}$$

$$9–11\;\mathrm{years},Y=-0.292\times^{2}+4.498X+12.647\;R^{2}=0.030\text{.}$$

$$12–14\;\mathrm{years},Y=-0.656\times^{2}+10.802X+0.881\;R^{2}=0.017\text{.}$$

$$15–17\;\mathrm{years},Y=-1.175\times^{2}+21.042X-44.185\;R^{2}=0.016\text{.}$$

Girls:


$$6–8\;\mathrm{years},Y=-0.160\times^{2}+2.544X+8.815\;R^{2}=0.025\text{.}$$

$$9–11\;\mathrm{years},Y=-0.331\times^{2}+6.657X-9.051\;R^{2}=0.009\text{.}$$

$$12–14\;\mathrm{years},Y=-0.509\times^{2}+12.200X-40.679\;R^{2}=0.048\text{.}$$

$$15–17\;\mathrm{years},Y=-0.537\times^{2}+9.313X-8.560\;R^{2}=0.010\text{.}$$

Total:


$$6–8\;\mathrm{years},Y=-0.188\times^{2}+3.121X+6.208\;R^{2}=0.027\text{.}$$

$$9–11\;\mathrm{years},Y=-0.284\times^{2}+5.049X+4.323\;R^{2}=0.013\text{.}$$

$$12–14\;\mathrm{years},Y=-0.483\times^{2}+9.723X-11.874\;R^{2}=0.006\text{.}$$

$$15–17\;\mathrm{years},Y=-0.765\times^{2}+13.778X-21.510\;R^{2}=0.007\text{.}$$

**Figure 3:**
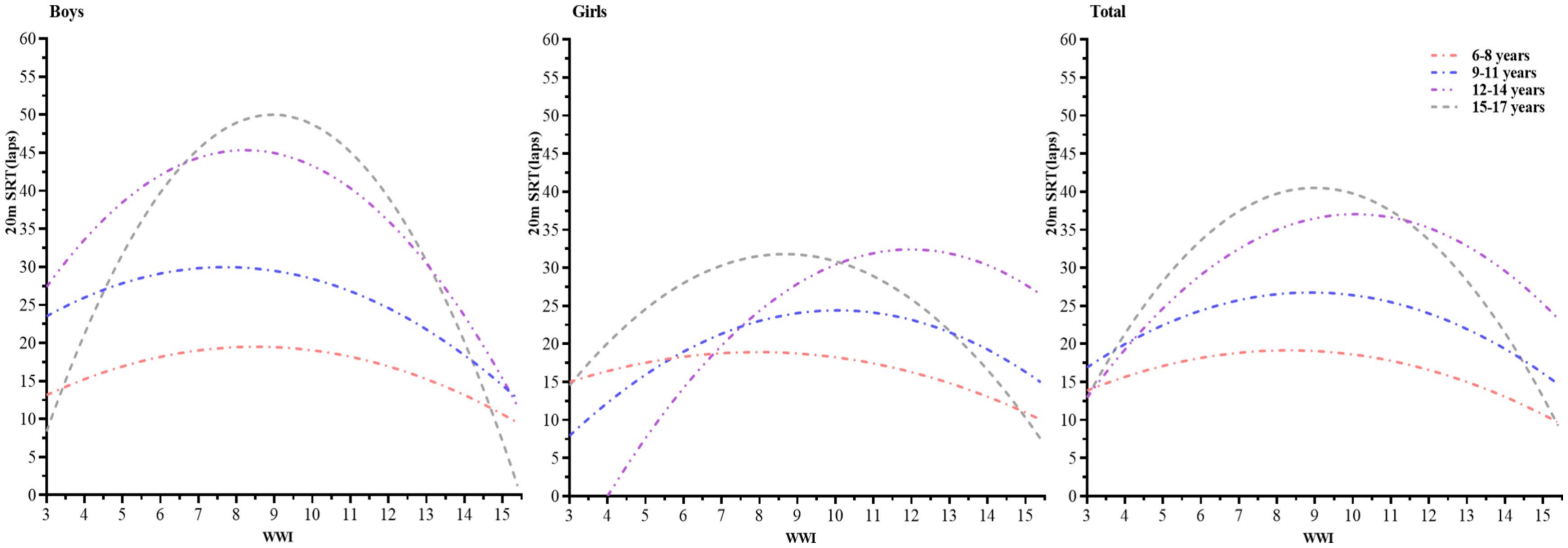
Trends in the performance of Chinese children and adolescents on the WWI and the 20-m SRT.

The graphs were plotted based on the curvilinear regression equations. Figure 3 shows the trend of WWI and 20-m SRT scores of Chinese children and adolescents. As can be seen from the figure, the relationship between WWI and 20-m SRT scores of Chinese children and adolescents shows an inverted “U” curve. The effect of WWI on the 20-m SRT was more pronounced in boys than in girls. In addition, the effect of WWI on 20-m SRT scores was more pronounced in the older age groups than in the younger age groups.

## 4 Discussion

To the best of our knowledge, this study is the first to use a nationwide sample from China to analyze the association that exists between WWI and 20-m SRT in children and adolescents. The results of this study showed that the 20-m SRT scores of Chinese children and adolescents aged 6–17 years showed an overall increasing trend with age, with the highest values in the 15–17 years age group. This finding is consistent with the conclusions of many domestic and international studies (2, 27). The 20-m SRT score mainly reflects cardiopulmonary fitness in children and adolescents. Cardiopulmonary fitness was found to show a gradual increase in childhood and adolescence as the cardiopulmonary organs matured, whereas in late adulthood it showed a Decrease in cardiopulmonary fitness (28). The results of the present study also showed that boys’ 20-m SRT levels were higher than girls’ in all age groups of Chinese children and adolescents, which is consistent with the findings of several past studies (29, 30). The reason for this is that boys and girls are genetically predisposed to higher levels of cardiopulmonary fitness due to their more developed skeletal muscles, which may be an important reason for higher 20-m SRT scores than girls. It has also been suggested that boys are more active by nature and participate in moderate to high-intensity physical activities for a longer period in their daily lives, which will inevitably lead to higher levels of cardiopulmonary fitness during exercise, and this may also be the main reason why boys’ 20-m SRT scores are higher than those of girls.

The results of the present study also showed that the differences in 20-m SRT scores between different WWI groups were statistically significant and that the overall 20-m SRT scores of children and adolescents in the 40th ≤ WWI <60th group had the highest WWI. This suggests that in the future, WWI should be kept at a moderate level in Chinese children and adolescents to ensure that cardiopulmonary fitness is at the highest level. Past studies have shown that WWI is an important indicator of body composition in children and adolescents, and there is a close correlation between an increase in WWI and several levels of physical fitness, with an increase in WWI reflecting an increase in body weight, which can lead to a decrease in physical fitness and level. Elevated WWI in the children and adolescents in this study implies an increase in body weight, and the assessment process of performing the 20-m SRT will inevitably require the participants to overcome a greater body weight resistance, which will lead to a decrease in 20-m SRT performance. It has also been shown that higher body weight individuals need to ingest more oxygen to maintain the body’s demand for oxygen during high-intensity cardiopulmonary fitness testing, thus placing higher demands on the cardiorespiratory system, whereas participants’ limited cardiorespiratory fitness is unable to safeguard the body’s exercise system’s demand for oxygen, leading to a decrease in exercise capacity, and thus increased body weight leads to a decrease in cardiorespiratory fitness level decreases. It is noteworthy that the relationship between WWI and 20-m SRT performance in this study showed an inverted “U” curve, i.e., low or high WWI led to a decrease in 20-m SRT performance. Lower WWI means lower body weight, and there is an association between lower body weight and muscle mass. Lower muscle mass during 20-m SRT will affect the level of muscular endurance and muscular strength, which is an important reason for the lower performance of 20-m SRT (31). Previous studies have shown that waist circumference and cardiopulmonary fitness are independently associated with indices of insulin resistance and glucose tolerance and mediate the association between moderate to vigorous physical activity and insulin resistance in an abdominally obese population (32). A survey of showed that the odds of alanine aminotransferase (ALT) > 30 increased 1.06-fold for each 1-cm increase in waist circumference, and there was a strong association between ALT levels and cardiopulmonary fitness, and a significant WWI-cardiopulmonary fitness association between WWI and cardiopulmonary fitness (33). It has also been shown that increased WWI may be closely associated with obesity-induced changes in gut flora, which can lead to mild inflammation in the body, which affects the body’s cardiorespiratory system and leads to lower 20-m SRT scores in those with higher WWI (34). It is evident that there is a strong correlation between WWI and 20-m SRT scores and that the reasons for this are multiple.

There are certain strengths and limitations of this study. In terms of strengths, on the one hand, to the best of our knowledge, this study is the first to use a national sample to analyze the association between WWI and 20-m SRT in Chinese children and adolescents, which provides a reference and helps to improve and intervene in cardiopulmonary fitness in Chinese children and adolescents. On the other hand, this study has a large sample size and involves participants from five major geographic regions in China, which makes the study sample representative. However, this study also has some limitations. First, the 20-m SRT was used to assess cardiopulmonary fitness in this study, and the 20-m SRT is an indirect measure. In the future, the cardiopulmonary exercise test (CPET), which is more objective, should be used to more accurately assess the relationship between WWI and cardiopulmonary fitness. Cardiopulmonary fitness. Secondly, this study should include corresponding covariates to more accurately analyze the association between WWI and cardiopulmonary fitness.

## 5 Conclusion

The relationship between WWI and 20-m SRT in Chinese children and adolescents has an inverted “U” curve, and this association was found in different genders and age groups. Both lower and higher WWI resulted in lower 20-m SRT scores, and the association was more pronounced in boys than in girls. In the future, Chinese children and adolescents should maintain a reasonable WWI value to ensure a high level of cardiopulmonary fitness and promote their physical and mental health. At the same time, the conclusion of this study provides a strong reference for health professionals, educational administrators and policy makers, and also provides help for effective intervention of cardiorespiratory fitness of adolescents.

## Data Availability

The raw data supporting the conclusions of this article will be made available by the authors, without undue reservation.
